# A population genomics approach to exploiting the accessory 'resistome' of *Escherichia coli*

**DOI:** 10.1099/mgen.0.000108

**Published:** 2017-04-06

**Authors:** Robert J Goldstone, David G E Smith

**Affiliations:** IB3, Heriot-Watt University, Edinburgh, UK

**Keywords:** antibiotics resistance evolution

## Abstract

The emergence of antibiotic resistance is a defining challenge, and *Escherichia coli* is recognized as one of the leading species resistant to the antimicrobials used in human or veterinary medicine. Here, we analyse the distribution of 2172 antimicrobial-resistance (AMR) genes in 4022 *E. coli* to provide a population-level view of resistance in this species. By separating the resistance determinants into ‘core’ (those found in all strains) and ‘accessory’ (those variably present) determinants, we have found that, surprisingly, almost half of all *E. coli* do not encode any accessory resistance determinants. However, those strains that do encode accessory resistance are significantly more likely to be resistant to multiple antibiotic classes than would be expected by chance. Furthermore, by studying the available date of isolation for the *E. coli* genomes, we have visualized an expanding, highly interconnected network that describes how resistances to antimicrobials have co-associated within genomes over time. These data can be exploited to reveal antimicrobial combinations that are less likely to be found together, and so if used in combination may present an increased chance of suppressing the growth of bacteria and reduce the rate at which resistance factors are spread. Our study provides a complex picture of AMR in the *E. coli* population. Although the incidence of resistance to all studied antibiotic classes has increased dramatically over time, there exist combinations of antibiotics that could, in theory, attack the entirety of *E. coli*, effectively removing the possibility that discrete AMR genes will increase in frequency in the population.

## Abbreviations

AMR, antimicrobial resistance; AMRF, antimicrobial resistance gene family; CARD, Comprehensive Antibiotic Resistance Database; HSP, high-scoring pair; SSS, sequence similarity score.

## Data Summary

1. Information on *Escherichia coli* genome sequences that we believe are mislabelled in GenBank has been deposited in Figshare; DOI: 10.6084/m9.figshare.4434776 (url – https://dx.doi.org/10.6084/m9.figshare.4434776).

2. A figure showing the phylogenetic position of genomes that we consider too distantly related to *E. coli* to be included in our analysis has been deposited in Figshare; DOI: 10.6084/m9.figshare.4434779 (url – https://dx.doi.org/10.6084/m9.figshare.4434779).

3. A list of genome sequences used in this study has been deposited in Figshare; DOI: 10.6084/m9.figshare.4434782 (url – https://dx.doi.org/10.6084/m9.figshare.4434782).

4. Our curation of the antimicrobial-resistance (AMR) determinants from the Comprehensive Antibiotic Resistance Database has been deposited in Figshare; DOI: 10.6084/m9.figshare.4434788 (url – https://dx.doi.org/10.6084/m9.figshare.4434788).

5. The sequence similarity score data underlying the analyses in our study has been deposited in Figshare; DOI: 10.6084/m9.figshare.4434794 (url – https://dx.doi.org/10.6084/m9.figshare.4434794).

6. A text-based representation of the graphs we used to generate antibiotic-resistance gene families has been deposited in Figshare; DOI: 10.6084/m9.figshare.4434797 (url – https://dx.doi.org/10.6084/m9.figshare.4434797).

7. A table of the genes we identified in the core resistome of *E. coli* has been deposited in Figshare; DOI: 10.6084/m9.figshare.4434800 (url – https://dx.doi.org/10.6084/m9.figshare.4434800).

8. A table highlighting the presence of core antibiotic-resistance determinants in the horizontally transferred genes of *E. coli* MG1655 has been deposited in Figshare; DOI: 10.6084/m9.figshare.4595371 (url – https://dx.doi.org/10.6084/m9.figshare.4595371).

9. A table of the resistance gene families we identified in the accessory resistome of *E. coli* has been deposited in Figshare; DOI: 10.6084/m9.figshare.4434803 (url – https://dx.doi.org/10.6084/m9.figshare.4434803).

10. A visualization of our data on the distribution of accessory resistance in *E. coli* has been deposited in Figshare; DOI: 10.6084/m9.figshare.4434809 (url – https://dx.doi.org/10.6084/m9.figshare.4434809).

11. A figure that shows it is unlikely that bias inherent in the sequenced *E. coli* resource has affected our analysis has been deposited in Figshare; DOI: 10.6084/m9.figshare.4595389 (url – https://dx.doi.org/10.6084/m9.figshare.4595389).

12. A figure that shows antibiotic resistance and phylogenetic distance are not correlated has been deposited in Figshare; DOI: 10.6084/m9.figshare.4595377 (url – https://dx.doi.org/10.6084/m9.figshare.4595377).

13. A visualization of the increase in the abundance of resistance to antibiotic classes in *E. coli* over time has been deposited in Figshare; DOI: 10.6084/m9.figshare.4434815 (url – https://dx.doi.org/10.6084/m9.figshare.4434815).

14. An animated visualization of the co-association of AMR in *E. coli* over time has been deposited in Figshare; DOI: 10.6084/m9.figshare.4434773 (url – https://dx.doi.org/10.6084/m9.figshare.4434773).

15. Data on the combinations of antibiotics for which all *E. coli* lack combined resistance has been deposited in Figshare; DOI: 10.6084/m9.figshare.4434818 (url – https://dx.doi.org/10.6084/m9.figshare.4434818).

## Impact Statement

Exposure of bacteria to antibiotics in clinical and veterinary settings has placed an immense selective pressure on bacteria that has accelerated the emergence of resistances. Using *Escherichia coli* as an exemplar, we identify the spectrum of accessory resistance determinants across the entire population of *E. coli* genome sequences available via the National Center for Biotechnology Information and profile the distribution of resistances. This profiling demonstrates the preponderance of multiple resistances among *E. coli* strains. Using this data, we model the potential for novel antibiotic combinations to modulate the emergence of resistant populations. These observations indicate that further consideration of population-level genome analyses should be incorporated into implementation of stratagems for managing antimicrobial-resistant pathogens.

## Introduction

The spread of antimicrobial resistance (AMR) in pathogenic bacteria is one of the key public-health concerns and national security risks of the modern era [1]. The diminution of efficacious treatments for infections has reached crisis point, not only in impoverished areas of the world, where mortality due to bloodstream infections by Gram-negative bacteria is more than double that caused by malaria [2], but also in affluent countries where bacteria with AMR are associated with many thousands of avoidable deaths each year [3, 4]. Resistance has emerged to almost every class of antimicrobial agent that has been developed [5], and the rapid evolution of resistance to these drugs has led to the fear of an end to the antibiotic era, and a return to a situation where even common ailments and injuries may be sufficient to kill [6].

Bacteria of the species *Escherichia coli* are among the leading causes of serious intestinal and extra-intestinal disease worldwide [7]. Of still greater concern, these bacteria are recognized as amongst the most resistant to antimicrobial agents that have a tradition of use in human or veterinary medicine [8]. *E. coli* demonstrates intrinsic resistance to a wide range of antimicrobial and toxic compounds, conferred by a combination of proteins that serve as multidrug efflux pumps, including TolC, AcrAB, AcrEF, EmrKY and MdtABC, amongst others [9–14]. Other mechanisms of resistance can include genetic polymorphisms that render the antibiotic's target less sensitive to inhibition [15–18]. However, high-level resistance to specific classes of antibiotics usually requires additional factors, including proteins that facilitate the export, modification or degradation of the antibiotic molecule [18].

The genes that confer AMR are found not only in isolates of human origin, but also in environmental bacteria, those isolated from domestic and wild animals [19–21], and even natural ecosystems or human populations secluded from modern medical or agricultural interventions [22, 23]. However, although AMR is present in diverse environments, the frequency of AMR can be strongly correlated with anthropogenic activity [24–26]. Furthermore, studies consistently demonstrate a trend for the increasing frequency of AMR and associated determinants over time [27–32].

Although the molecular mechanisms of many AMRs are well characterized, investigation of the population genetics of antibiotic resistance within the *E. coli* species has received less attention. Understanding the diversity and distribution of AMRs in this species is crucial to understand the evolution of AMR and to explore whether this information can help us extend the useable lifespan of antibiotics in the absence of novel discoveries.

## Methods

### Acquisition, curation and phylogenetic analysis of *E. coli* genome sequences

We downloaded the nucleotide sequences of 5788 *Escherichia* and *Shigella* genomes from GenBank on the 19th October 2016. Source and date of isolation data and reference information available in the GenBank record was collected at the same time. Four strains were excluded because the strain names and BioSample accession numbers (used for indexing the strains) failed to be retrievable. We selected good quality genome sequences from these records based on the following criteria: (1) sequence contained fewer than 0.1 % ambiguous bases (maximum approximately 5000 ambiguous nucleotides specified by the character), (2) assembly comprised fewer than 500 contigs, (3) greater than 3 Mb and less than 7 Mb sequence length (smaller genomes tended to be incomplete, and larger genomes tended to be mixed samples comprising more than one bacterial genome). By applying these criteria, we identified 4084 genomes that we considered good quality. Next, we queried these genomes to ensure they belonged to genuine genus *Escherichia* genomes. To do this, we probed these genomes for the presence of 4322 gene sequences from the reference *E. coli* K12 strain MG1655 (U00096) genome using blast [33], and excluded 11 genomes that shared fewer than 2000 genes in common with this sequence. Aside from the excluded sequence for *Escherichia vulneris*, the remainder of the genome sequences we excluded we suspected were entirely, or at least heavily contaminated with, non-*Escherichia* or *Shigella* DNA (see Data bibliography 1).

To determine which among the remaining 4073 sequences represented true species *E. coli* genomes, we used our data to determine sequences that would be useful for phylogenetic reconstruction of the population structure of genus *Escherichia* and *Shigella* genomes. We identified 16 genes that could be reliably recovered from the genomes of all 4073 strains, 80 genes that could be recovered from the genomes of at least 4072 strains, and 201 genes that could be recovered from the genomes of at least 4071 strains. Subsequently, we extracted the nucleotide sequences for the 201 genes, aligned this individually using Muscle [34], concatenated them to one sequence, removed poorly aligned regions using Gblocks [35], and reconstructed a maximum-likelihood tree under the GTR model using RaxML [36]. The resulting tree was investigated to identify branches that fell outside the major *E. coli* lineages (for example strains more closely related to *Escherichia albertii* or *Escherichia fergusonii*) – 51 strains were removed from further analysis (supporting data under Data bibliography 2 shows the position of these genomes relative to *E.* coli). This resulted in a final population of 4022 *E. coli* and *Shigella* sequences that were used in further analysis. *E. coli* phylogroups were assigned by grouping genomes into the largest monophyletic group that included all known target phylogroup members, whilst excluding all others. Within the GenBank records, strains isolated from similar sources can have different annotations (i.e. human faecal and *Homo sapiens* stool). To interrogate the source of isolation for each strain, we reduced the disparate annotations to the shortest list we could devise, using the isolation source data and the reference information in the GenBank record, alongside our own knowledge of widely used reference strains where this information was missing in the GenBank record. Our final source of isolation classification is presented alongside the list of genomes used in this study in Data bibliography 3.

### Curation of antibiotic-resistance factors

Data from the Comprehensive Antibiotic Database (CARD) [37] was downloaded on the 19th October 2016. The antibiotic resistance ontology (ARO) designation for each gene present in the CARD homologue model was cross-referenced with the ARO index to determine the resistance profile and mechanism for each gene. We supplemented these resistance profiles with information for resistance to olaquindox as resistance determinants (*oqxA, oqxB*) were present in the CARD, yet the antimicrobial was not. Other supplementation included assigning ‘multiple drug’ classifications to known mediators and regulators of multidrug efflux. Our supplementary curation is provided in supporting data under Data bibliography 4.

### Determining the presence of the CARD determinants within the *E. coli* population

We queried the presence of the genes contained in the protein homologue model of the CARD within the *E. coli* population by blast. To recover sequences possibly split across contigs, we recovered high-scoring pairs (HSPs) that matched the reference sequence by greater than 80 % identity, where the database sequence covered more than 40 % of the query sequence. If more than one HSP for a given query sequence was recovered from the database sequence, the query sequence participating in the HSP was mapped against the full-length gene, and this mapping used to reconstruct the full-length gene from the database sequence participating in the HSPs. The mean identity of HSPs participating in the mapping and the percentage coverage of the database sequence over the query sequence was used to calculate a sequence similarity score (SSS), which is the mean of sequence identity and the percentage sequence coverage. A matrix of hits we identified in *E. coli* is provided in supporting data under Data bibliography 5 – these data underlie our analyses. We defined a gene as being present in a genome when the query sequence matched within the genome at a SSS of greater than 80 %. We then assigned the matched genes into two groups: core determinants – genes present in more than 95 % of the *E. coli* population; and accessory determinants – genes present in fewer than 95 % of the *E. coli* population. We eliminated four genes from the accessory determinants: (1) the gene encoding the pesticin receptor *fyuA*, as we could not find any primary literature to indicate this gene played a role in AMR; and (2) the genes *mdtM*, *ermA* and *pmrE* from MG1655. We excluded these latter genes as MG1655 is generally considered sensitive to antibiotics and so we presumed these are unlikely to confer high-level resistance.

### Further treatment of the accessory determinants

To ensure we had accurately determined accessory resistance elements rather than related orthologues that might not provide resistance, we purified the genes we had assigned to be present in the accessory genome by requiring them to have a SSS of 98 % or greater. This resulted in the identification of 1029 homologues within the accessory determinants. Since many of the determinants in the CARD are closely-related factors with slightly modified specificities (for example TEM family β-lactamases), we decomposed the accessory resistance determinants into AMR gene families (AMRFs). To do this, we extracted the genes of the accessory resistance determinants from the CARD and, using blast, retrieved a list of gene pairs where those genes had a SSS of 95 %. We examined these gene pairs to ensure each paired AMR determinant conferred resistance to the same profile of antibiotics. In cases where the AMR profiles differed, the pairs were excluded from the network – these were confined to pairs between AAC(6')-Ib-cr (fluoroquinolone and aminoglycoside resistances) and other similar AAC(6') genes that confer resistance only to aminoglycosides [38]. Remaining gene pairs were then passed to the graph building algorithm MCL [39] to build gene families containing networks of homologues. We used these gene families to collapse the accessory resistance determinants for each strain to one call per family – if a strain matched at least one gene in the family network to at least 98 % we called this gene family as present in this strain. The networks we used to generate the AMRFs are presented in supporting data under Data bibliography 6.

### Statistical evaluation of AMR carriage

To investigate whether the number of AMRs found in genomes was different to what would be expected if AMR determinants were randomly distributed in genomes, we employed a resampling algorithm. Here, we randomized the distribution of AMRFs over 100 000 replications, and counted the number of resistances these families provided to each strain. We then compared the actual observed number of resistances with this null distribution. Significance was set at 0.0001.

To test for the possibility of bias within the genome sequences – caused by the selection of strains for sequencing based on their clinical importance, or indeed their antibiotic-resistance profile – first we investigated whether the sequenced genomes in the National Center for Biotechnology Information database reasonably represented the diversity of *E.* coli; that is to say, how closely related could a newly sequenced isolate of *E. coli* be expected to be to an existing genome in the sequenced database. To do this, we calculated a pairwise distance matrix from the distances between the tips of the phylogenetic tree using the ‘cophenetic.phylo’ function within the ape package [40] in R. We then randomly drew strains from the list of tree tips over increasing sample sizes (from 2 to 4022), took one of these randomly drawn genomes (a proxy for a newly sequenced strain) and recorded the distance between this sequence and the most closely related genome from the sample (a proxy for existing genomes in the database). We repeated this for 10 000 replications per sample size. Next, to investigate whether the source of isolation of *E. coli*, or the fact that some genomes have been sequenced because of their AMR profile, overly affected our analysis, we employed several variations of our AMR resampling method on samples of genomes isolated from different sources: all strains listed as isolated from non-human and non-unknown sources; all strains where the words ‘antibiotic’, ‘antimicrobial’, ‘resistance’, or ‘resistant’ did not appear in the reference title; all strains listed as from ‘human.urine’, ‘human.blood’, or ‘human.bodyfluid’; all genomes listed as isolated from ‘farms’, ‘cows’, ‘avian’, or ‘sheep’.

### Testing the relationship between clonal-lineage and AMR carriage

To investigate the possibility that phylogenetic distance between strains could be correlated with AMR gene carriage, we calculated two distance matrices – one from the phylogenetic tree describing the population structure of *E. coli*, and one from the distribution of AMRFs. We then sampled 100 000 identical pairwise distances from these matrices, and compared the phylogenetic distance with the AMR distance calculated for each pair. To investigate whether clonal or lineage-related groups of *E. coli* tended to be more enriched for AMR genes than more disparate strains, we split the population structure of *E. coli* into two groups – lineage-related strains or non-lineage-related strains – based on the following criteria: (1) lineage-related strains existed on subtrees with 10 or more tips, and (2) the maximum distance observed between any two genomes of the subtree was no greater than 1 % of the maximum distance observed between all *E. coli*. These criteria split the population of *E. coli* into two roughly equal groups. We then repeated our resampling experiment for the evaluation of AMR gene carriage for these two groups.

### Estimation of significantly associated resistances

To investigate which antibiotic combinations were found in strains more frequently than would be expected by chance, we used our resampling algorithm. In this case, the AMRFs for the population under investigation (the whole population for ‘all strains’, or the genomes isolated up until and including the stated year for our time-course analysis), we randomized over 10 000 replications. At each iteration, for each pair of AMRs, the number of genomes encoding AMRFs directed at these pairs of AMR were counted. The actual observed number of co-resistances in the population was then compared with this null distribution. We considered a pair of resistances to be significant if less than two of the randomizations yielded a count as large or larger than the actual value. These relationships were visualized using the igraph package [41] within R.

### Modelling

To simulate the effect of applying antibiotic combinations on a hypothetical bacterial population, we designed the following model. Firstly 100 random genomes were selected from the *E. coli* population, and the distribution of AMRFs in these genomes recorded. For 1000 generations, the following steps were followed. (1) One random AMRF was chosen for a chance to spread – if the population contained at least one strain with the chosen AMRF, a randomly selected *E. coli* was donated this determinant. If the population did not contain the AMRF, nothing changed. If the randomly selected *E. coli* already has this determinant, nothing changed. (2) A randomly selected *E. coli* was selected, which was then 'exposed' to the specified combination of antibiotics. If the genome was sensitive to any of the specified antibiotics, the genome was removed from the population. If the genome was resistant to all specified antibiotics, it was placed back into the population. (3) If the strain was removed from the population, a randomly selected genome was duplicated in the population, or if the strain was returned to the population, this genome was duplicated. At each generation, the number of AMRFs in the population and the size of the population relative to the start values was recorded. For the simulations, the model was run for 1000 replicates per antibiotic combination.

## Results

### Core resistome of *E. coli* is primarily composed of multidrug efflux pumps

The core resistome of *E. coli* (defined as: 1, homologues of greater than 80 % identity with the reference sequence present in the CARD; and 2, present in 95 % or more strains) comprises 50 genes (shown in supporting data under Data bibliography 7). Although we expected these genes to be fixed within *E. coli* genomes and not to be subject to horizontal gene transfer (HGT), we were surprised to find six (*evgA*, *evgS*, *emrK*, *emrY*, *gadE* and *gadX*) listed as putatively horizontally transferred genes in the HGT-DB [42] (Data bibliography 8).

However, we are not clear that all the genes listed in the CARD mediate antibiotic resistance in *E. coli*. Within species such as *Streptococcus pneumoniae*, *patA* encodes part of an efflux pump that has been shown to export fluoroquinolones [43]. However, in *E. coli*, *patA*, under the accession number listed in the CARD (NP_417544.5), encodes a putrescine-2-oxoglutaric acid aminotransferase enzyme and we could not find any literature describing this gene relating to AMR in *E. coli*. Similarly, for the *mfd* gene, which putatively confers resistance to fluoroquinolones in *Campylobacter jejuni* [44], we could find no literature describing a similar role in *E. coli*. It is also uncertain that the genes *cysB* and *alaS* influence aminocoumarin resistance in the core resistome of *E. coli*. Variants in both these genes can cause reduced sensitivity to novobiocin [45], and it is possible that our identity criteria have not discriminated the aminocoumarin-resistant variants from their more sensitive counterparts. However, the SSS for these two genes in our analysis tended to be very high, and *E. coli* has previously been suggested to be intrinsically insensitive to aminocoumarins [46].

Except for these entries, the remaining genes in the core resistome mediate or modulate the efflux of multiple drugs, including the *acr*, *mdt* and *emr* genes [12]. Other genes in the core resistome included the *pmr* genes, which confer minor alterations to the structure of cell surface lipopolysaccharide and reduce sensitivity to polymyxin class antibiotics, and *bacA*, which mediates resistance to peptide antibiotics such as bacitracin.

### Accessory resistome of *E. coli* is non-randomly distributed

Within the CARD homologue model sequences, there were 90 AMRFs that matched to sequences found in the *E. coli* population (shown in supporting data under Data bibliography 9). These mediate resistances to 18 classes of antibiotic. Table 1 summarizes a selection of reported MICs for certain *E. coli* strains. These resistances around the phylogenetic tree of *E. coli* are shown in supporting data under Data bibliography 10.

**Table 1.** Accessory antibiotic resistance in *E. coli* This table lists 14 antibiotic classes to which *E. coli* has been reported to be sensitive, and the number of AMRFs from the accessory resistome that are active against each of these classes. These antibiotics are abbreviated as follows: KM (kanamycin), SM (streptomycin), NEO (neomycin), GEM (gentamicin), AMP (ampicillin), VQC (vancomycin-QC_14_), CAM (chloramphenicol), FOS (fosfomycin), CLIN (clindamycin), ERM (erythromycin), OLA (olaquindox), FLOR (florfenicol), HFU (81.723 hfu), COL (colicin), NAL (nalidixic acid), CIPRO (ciprofloxacin), NOR (norfloxacin), RIF (rifamycin), STRG (streptogramin G), STRF (streptothricin F), NOUR (nourseothricin), SUL (sulfamethoxazole), TET (tetracycline), TRI (trimethoprim).

Antibiotic class	No. of AMRFs	Typical mode of action	Reported MIC (µg ml^−1^)	Reference
Aminoglycosides	20	Protein synthesis inhibitors	2 (KM) 1.95 (SM) 1 (NEO) 0.2 (GEM)	[9] [81] [81] [81]
β-Lactams	23	Peptidoglycan biosynthesis inhibitors	12.5 (AMP)	[9]
Glycopeptides	1	DNA damaging agents	4.5 (VQC)	[82]
Chloramphenicols	6	Protein synthesis inhibitors	6.25 (CAM)	[9]
Fosfomycins	2	Peptidoglycan biosynthesis inhibitors	32 (FOS)	[83]
Lincosamides	2	Protein synthesis inhibitors	100 (CLIN)	[84]
Macrolides	8	Protein synthesis inhibitors	50 (ERM)	[9]
Olaquindox	2	DNA synthesis inhibitor	9 (OLA)	[85]
Phenicols	1	Protein synthesis inhibitors	4 (FLOR)	[86]
Pleuromutilin	1	Protein synthesis inhibitors	1 (HFU)	[87]
Polymyxin	1	Membrane disruption	0.5 (COL)	[82]
Quinolones [Q]/fluoroquinolones [F]	7	DNA damaging agents	3.125 (NAL) [Q] 0.01 (CIPRO) [F] 0.004 (NOR) [F]	[9] [9] [9]
Rifampin	1	RNA synthesis inhibitors	2.4 (RIF)	[88]
Streptogramin	3	Protein synthesis inhibitors	500 (STRG)	[89]
Streptothricins	1	Protein synthesis inhibitors	8 (STRF) 2 (NOUR)	[81] [81]
Sulfonamides	3	Dihydropteroate synthetase inhibitors	8 (SUL)	[90]
Tetracyclines	4	Protein synthesis inhibitors	1.25 (TET)	[9]
Trimethoprim	14	Dihydrofolate reductase inhibitor	2 (TRI)	[91]

**Fig. 1.**
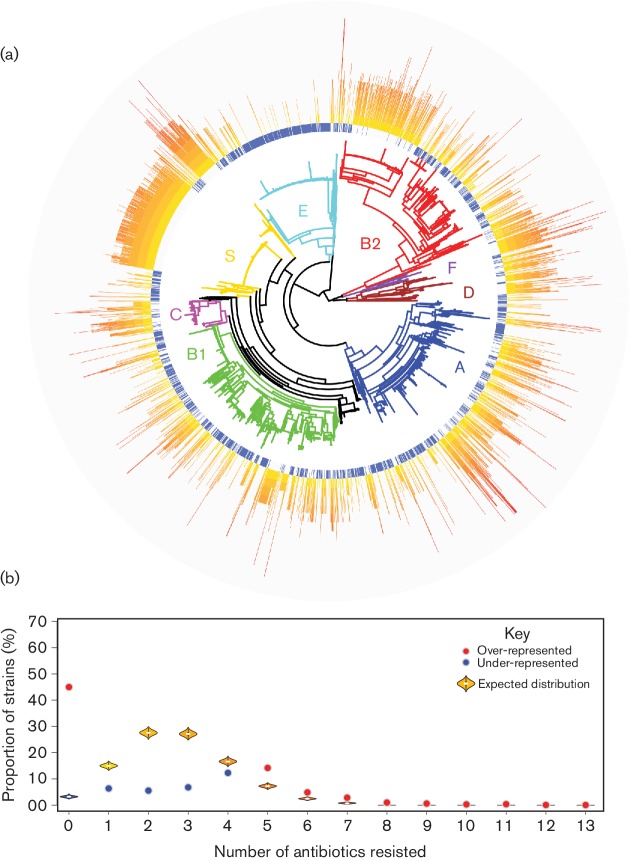
The abundance of antibiotic resistance in the *E. coli* population. (a) The distribution of the abundance of accessory AMR around the *E. coli* population. Clearly there is a wide diversity of AMR, with high-level resistances found in most phylogroups, with particularly high concentrations of resistance in phylogroups C, D and F. Phylogroup E (mainly O157 : H7) displays remarkably low levels of accessory resistance. (b) A violin plot for the strains that would be expected to encode the specified number of AMRs if the distribution of resistance genes was randomly distributed amongst the genomes. This can be contrasted with the observed number, represented as a coloured point. An observed number significantly greater than the expected distribution is represented as a red point, whereas an observed number significantly lower than the expected distribution is represented as a blue point (*P<*0.0001).

Different *E. coli* strains encode multiple different AMR determinants – a fact clearly illustrated in Data bibliography 8 and 9. We were interested in exploring how the distribution of these AMRs translated to the total number of antibiotics each strain was capable of resisting, and so we calculated the number of separate AMRs conferred by the complement of determinants present in each strain (Fig. 1a). These data revealed that AMR was not evenly spread across the *E. coli* population, with a large proportion of strains within phylogroups C, D, F and *Shigella* encoding resistance, while resistance in phylogroups A, B1 and, to a lesser degree, B2 was more sporadic. Phylogroup E strains were marked by a very low level of AMR. Clearly, there are many genomes that encode resistance to a considerable number of antimicrobials. To explore whether the proportion of genomes conferring resistance to different numbers of antibiotics was unusual, we performed a re-sampling analysis whereby, assuming the distribution of resistance determinants was randomly assorted between the genomes, we could calculate how many strains we would expect to encode increasing numbers of AMRs, and compared this with the observed number of strains (Fig. 1b).

These data showed that the numerical abundance of AMR in *E. coli* clearly deviated from the range we would expect to find if resistance determinants were randomly distributed between the genomes – this expected distribution is shown as violins within Fig. 1(b). The observed value is shown as a coloured point: a red point indicates an observed value significantly greater than expected, whilst a blue point represents an observed value significantly lower than expected. We were also particularly surprised to find such a large proportion of genomes to encode no accessory resistance determinants – almost 45 % (1809 genomes). This was considerably more than if determinants were randomly distributed across strains, where we would expect on average only 232 strains to lack AMR determinants. By contrast, the number of strains encoding one, two, three or even four AMRs was significantly less than would be expected by chance. However, as the number of resistances rose to five or more, we observed a shift in the significance, whereby the number of strains we observed in these groups was significantly greater than chance – in most instances dramatically so. If resistance determinants were randomly distributed through *E. coli* we would expect, on average, just 169, 37, 6 and 1 genomes to contain five, six, seven or eight AMRs, respectively, and no strains would encode resistance to nine or more AMRs. However, we observed that 570 genomes encoded five AMRs, 195 encoded six AMRs, 114 encoded seven AMRs, 39 encoded eight AMRs, 24 contained nine AMRs, 11 contained ten AMRs, 14 contained eleven AMRs, 2 contained twelve AMRs and even 1 genome encoded resistance to thirteen separate classes of AMR.

Given the sampling of *E. coli* selected for sequencing is not random, we investigated the possibility that the loading of the sequence database with clinically important strains, for example from human blood or urinary tract infections, had biased our analyses. To do this, we explored several avenues. Firstly, we found that the samples of *E. coli* that have been sequenced, although not selected in a random fashion, likely saturate the total diversity of *E. coli*. In Data bibliography 11(a), we show that a newly sequenced *E. coli* will, on average, be a phylogenetic distance of just 0.0009 from another, already sequenced isolate. To put this distance in context, this is comparable with the distances calculated between several sequenced O157 : H7 genomes, and only slightly greater than the distances calculated between sequences for different K-12 clones. The relative position of this distance value in an ordered list of unique distance values calculated from the tree is shown in Data bibliography 11(b). This observation may mitigate the effect of a biased selection of strains for sequencing, as it indicates against the possibility that sequenced *E. coli* represent particularly AMR-enriched sub-lineages within the wider population structure of *E. coli*, which are otherwise unexplored. Secondly, in Data bibliography 11(c), we could show that the results of our analysis did not change substantially by removing all known *E. coli* isolated from human (and unknown) sources from the analysed population, nor by removing all genomes for which the reference given in the GenBank record contained words related to antibiotic resistance. Furthermore, by limiting our analysed population to strains isolated from sources that were likely to be considered clinically important, such as human blood or urine, or which were unlikely to be considered clinically important, such as those from agricultural land or farmed animals, we still saw these groups had significantly more genomes with no accessory resistance than would be expected if AMR genes were randomly assorted between strains, along with significantly more strains having large numbers of AMR genes within their genomes, regardless of isolation source.

In some other species of bacteria, AMRs are concentrated into specific clonal or lineage-associated groups [47]. However, even though, superficially, some particularly highly sampled clonal groups of *E. coli*, such as *Shigella* or O157 : H7, shared similar profiles of AMR, we could not detect a relationship between phylogenetic distance and similarity in AMR gene carriage (Data bibliography 12(a)). To further test the possibility that clonal or lineage-related strains tended to encode high-levels of AMR genes, we separated the population structure of *E. coli* into two groups – lineage-related strains and non-lineage-related strains – based on the following criteria: (1) lineage-related strains existed within subtrees of the larger phylogenetic tree that contained ten or more strains, and (2) where the maximum distance between any two of those strains was less than 1 % of the maximum distance between any *E.* coli. The resulting trees reconstructed from these two groups can be seen in Data bibliography 12(b). As shown in Data bibliography 12(c), when we analysed these strains for significant enrichments in AMR gene carriage, the profiles were remarkably similar for both groups as we observed for the whole population. Together, these data indicated that phylogenetic relatedness between *E. coli*, perhaps surprisingly, does not play a large role in determining the spectrum of AMR genes an individual strain harbours, and furthermore, the highly resistant strains are not concentrated within specific and closely related clonal, or lineage-related, groups.

### Incidence of multidrug resistance in the *E. coli* population has increased over time

**Fig. 2.**
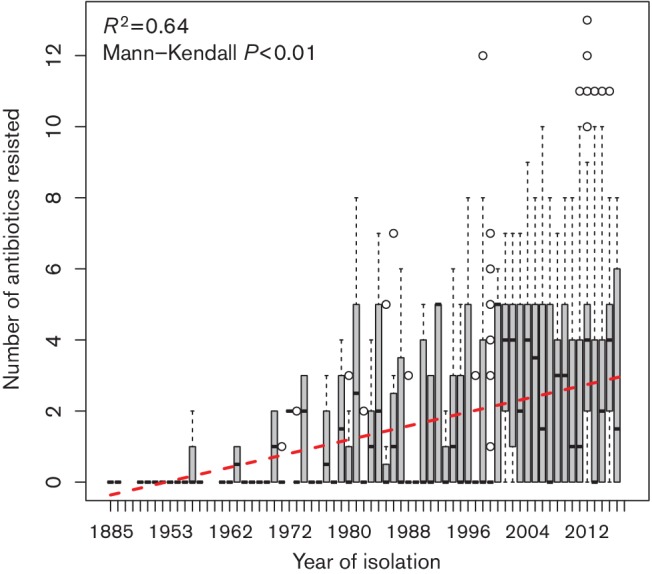
Temporal increase in multi-drug resistance in *E. coli*. Sequenced *E. coli* have been collected over time, spanning from 1885 to the present day. The number of antibiotics resisted in strains collected in successive years shows a strong increase over time (Mann–Kendall test *P*<0.0001). The red dotted trend line is fitted from a linear model (*R* ^2^=0.64).

Given that the number of AMRs present in *E. coli* appears to follow a non-random distribution, we speculated that the trend towards concentration of AMRs may be preserved in the genomic 'fossil record'. From the 4021 genome sequences in our panel, 2925 (72.7 %) of these had their year of isolation recorded in the GenBank record – spanning years between 1885 to 2016. We used this information to count the number of AMRs in strains isolated from the same year, and plotted this data in the box-plot shown in Fig. 2. These data revealed a clear positive correlation between successive years’ isolations and increasing AMRs. Investigation of this trend via the Mann– Kendal test revealed a significant trend towards increasing AMR in isolates over time. In fact, not only were the total number of AMRs found in isolates increasing over time, but also each AMR detected has similarly increased (shown in the supporting data under Data bibliography 10).

### AMR in *E. coli* is highly interconnected

The concentration of AMR within *E. coli* strain leads inevitably to the situation whereby treatment of an infection with one antibiotic will probably – if the population of *E. coli* includes a resistant strain – result in the concomitant population increase in the level of resistance to other, unused antibiotics. This assumption led us to question whether we could detect which antibiotic classes were likely to be encoded together. To do this, we constructed a network of all the co-resistance phenotypes found in the *E. coli* population. Then, to determine which edges of this network were significant, we randomized the distribution of AMRFs over 10 000 replications and eliminated the edges where at least one of the randomizations resulted in a number of genomes containing both resistances which was as large as – or larger than – the observed value (equivalent to *P* *=*0.0001). This analysis revealed a network of AMR genotypes in *E. coli* that were highly interconnected. Indeed, all 18 classes of AMR found in *E. coli* were found more often with at least one other class of AMR than would be expected by a random distribution of AMR genes. β-Lactam resistance was the most highly connected AMR, and was significantly associated with 12 other AMR phenotypes, whilst other highly connected AMR phenotypes included trimethoprim (11 connections), sulfonamide (9 connections), aminoglycoside (8 connections) and chloramphenicol (7 connections). A visualization of how this network of antibiotic resistance has evolved over time is presented as an animated picture in supporting data under Data bibliography 14.

### Combination of antibiotics reduces the microevolution of resistance in a computer model

**Fig. 3.**
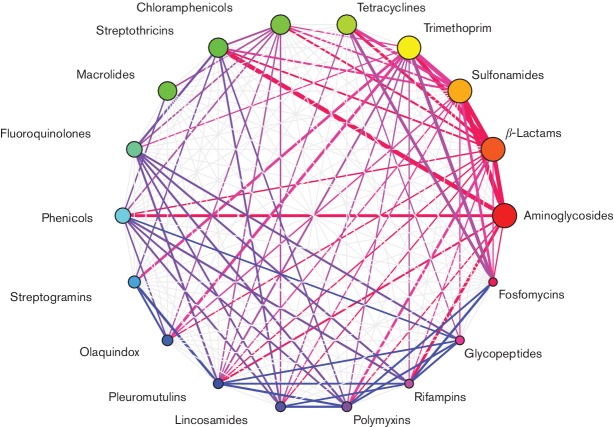
The highly-interconnected network of antibiotic resistance in *E. coli.* This figure shows a network of AMRs that are more frequently found together in *E. coli* than would be expected if AMRFs were randomly distributed across genomes. The vertex sizes are proportional to the number of strains that encode the resistance, while the edge widths are proportional to the number of strains that encode both the connected vertices as a function of how many contain either. Edges are coloured for clarity of visual representation of connections .

As implied in Fig. 3, the use of any antibiotic will likely, when the population contains resistant strains, result in an increase in the level of resistance to multiple antibiotics. What may be needed, therefore, to combat the rise of resistance is to use knowledge of the diversity of *E. coli* and formulate combinations of antibiotics which, when used together, should be sufficient to kill any *E. coli*. One of the most pernicious problems with the use of antibiotics is that the effect of the antibiotic is not localized, but is distributed to all resident microbiome bacteria. *Ergo*, if any of these bacteria encodes resistance to the antimicrobial used, the result will be an increase in the population level of AMR, even if an infection is resolved following the use of antibiotics. By investigating the diversity of AMR in *E. coli*, we have estimated combinations of three antimicrobials that theoretically should kill all *E. coli –* with the assumption *E. coli* are all drawn from the known population (Data bibliography 15). Unsurprisingly, many of these combinations involve those antibiotics where resistance is relatively rare in *E. coli*.

**Fig. 4.**
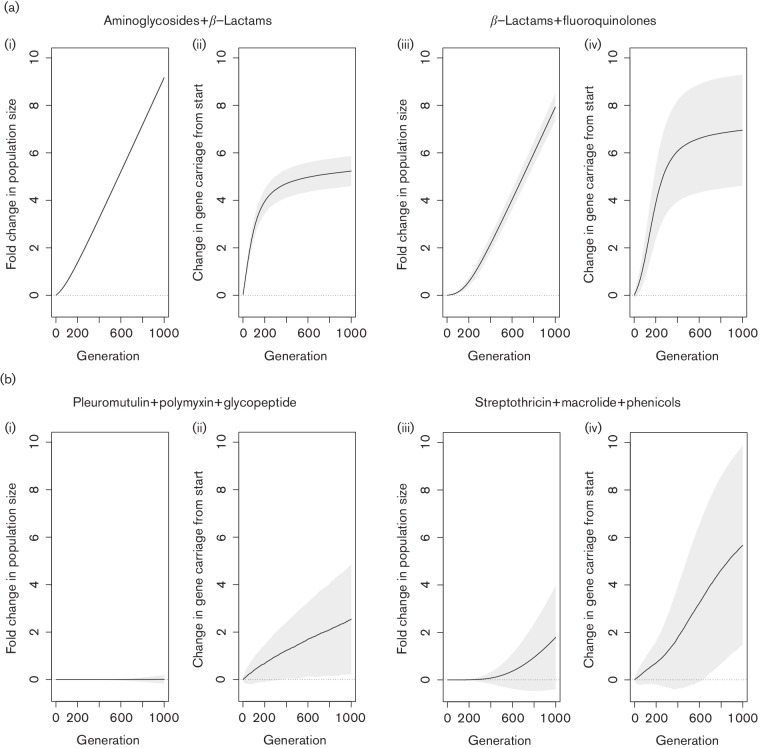
Simulated effects of antibiotic combinations on population growth and AMR spread. This figure shows the results of our simulation for commonly used antibiotic combinations (a), and the best [b(ii) and (iii)] and worst [b(iii) and (iv)] performing combinations from our analysis of antibiotic combinations that can kill any *E. coli*. For commonly used combinations, the population in our simulation rapidly expanded [a (i) and (iii)], and the mean number of resistance determinants in the population quickly increased [a(ii) and (iv)]. For the best performing antibiotic combination in our model (pleuromutilin with polymyxin and glycopeptide), population expansion was minimal [b(i)], while the rate of spread of AMR was low [b(ii)]. For the worst performing combination in our model (streptothricin with macrolide and phenicols), we observed a twofold population increase over 1000 generations [b(iii)], while the mean number of resistances in the population increased substantially [b(iv)].

To demonstrate this effect in a simple simulation, we constructed a model to investigate how the application of antibiotic combinations could change population dynamics in respect of both bacterial and AMR gene numbers. Our model had simple parameters using a sample of strains collected at random from the *E. coli* population. At each generation, one gene family was randomly selected for the chance to spread. So long as that gene was present in the sampled population, and since our data indicated that antibiotic resistance tends to co-associate, we selected the recipient strain randomly from the subpopulation that already encoded at least one resistance gene. One strain was then chosen as being exposed to a combination of antibiotics. If the strain was resistant to all the selected antibiotics, it was placed back into the population and its genotype was replicated. If the strain was sensitive to at least one antibiotic, it was removed from the population, and a randomly selected genome was chosen to replicate (to prevent the population crashing to zero). We also added an element of openness by allowing, in each generation, a chance for one strain to capture a random AMR gene from outside the population. We linked this chance with the size of the population, so that larger populations had an increased chance of capturing novel genes. At each generation, the mean number of resistance genes found in the population was recorded and calculated as the number of additional resistance determinants in the population compared with the start of the experiment. We also kept track of the number of bacteria in the population, expressed as a fold change from the original population (Fig. 4).

Fig. 4(a) shows the consequence of two of the most frequently employed combinations of antibiotics – aminoglycosides with β-lactams, and β-lactams with fluoroquinolones – in our model. Since resistance to these antibiotics are so commonly observed together, resistant strains are frequently drawn from the population. This causes the population to quickly grow [Fig 4a (i, iii)]. Furthermore, and likely because these resistances are frequently encoded alongside several other resistances, the mean number of resistances in the population also rapidly expands [Fig 4a (ii, iv)]. The number of antibiotic-resistance genes expands to potentially greater numbers in the β-lactam/fluoroquinolone treatment [Fig. 4a (iv)] than in the aminoglycoside/-lactam treatment [Fig. 4a (iii)].

This can be contrasted with selecting combinations of antibiotics that our previous results indicated should in theory kill any *E. coli*. We ran our model using all 118 combinations shown in supporting data under Data bibliography 12 to investigate the combinations – per our model parameters – that resulted in the most efficacious killing and the slowest rate of spread of antibiotic-resistance genes (results are shown alongside the combinations used in Data bibliography 12. Our results showed that some combinations of these antibiotics should be more efficacious than others (Fig. 4(b), with one of the most efficacious combinations – pleuromutilin with polymyxin and glycopeptides – outperforming the least effective by a considerable margin in terms of suppressing population growth [compare Fig 4b (i, iii)] and reducing the rate of the spread of resistance genes [compare Fig. 4 b (ii, iv)]. In fact, exposure to pleuromutilin, polymyxin and glycopeptide led to only a marginal population increase over the 1000 generations, indicating the rate at which *E. coli* gained resistance to all three of these antibiotics in our model was very slow. This was observed alongside the lowest rate of AMR determinant increase for any combination. This is in contrast with the streptothricin, macrolide and phenicol combination, which resulted in at least a twofold increase in the population size at the end of 1000 generations [Fig. 4b (iii)], and approximately six more resistance determinants per strain [Fig. 4b (iv)].

## Discussion

Our analyses indicate that all *E. coli* encode a suite of core resistance genes that presumably facilitate basal levels of non-specific resistance to a variety of antimicrobial compounds. The factors encoded within the core resistome tend to be multidrug efflux pumps and regulators that likely enable *E. coli* to navigate through environments that contain low levels of a wide variety of toxic molecules, including antibiotics produced by co-resident bacteria and fungi, dyes, free fatty acids, and antimicrobial compounds produced by eukaryotic hosts including bile salts [12–14, 48–50], or even endogenously produced toxic by-products of metabolism [51, 52]. These observations support the assertions put forward by others that the intrinsic resistance of bacteria is due to the activity of multidrug efflux pumps [13, 14].

In addition to the core resistome, many, but not all, *E. coli* encode accessory resistance determinants. Intriguingly, and despite the perception that *E. coli* represent amongst the most resistant bacteria to antibiotics commonly employed in medical and veterinary medicine [8], accessory resistance factors are not as frequent in *E. coli* strains as may be expected, and only slightly more than half of all *E. coli* encode detectable accessory resistance factors. Phylogroup E isolates tend to encode by far the fewest AMRs and, hence, tend to be resistant to few antibiotic classes. This fact may be explained, at least in part, by phylogroup E representing largely a homogeneous group of *E. coli*, with a large majority of these isolates drawn from the O157 : H7 genotype, which, it could be argued, is massively oversampled. The general lack of AMRs in the O157 : H7 cluster is contrasted with phylogroup E strains that fall outside this group, such as the O157 : H16 strain Santai (BioSample accession no. SAMN02673556), which encodes resistance to 11 antibiotic classes. Nevertheless, the low abundance of antibiotic resistances in this serotype has also been detected in previous surveys [53]. The low level of resistance in this group of *E. coli* is accompanied by the knowledge that antibiotic treatment of O157 : H7 infections is known to worsen the disease [54]. However, it is difficult to imagine how this facet alone would lead to a strategy that is evolutionarily successful. Other studies have, however, detected modest [55–57] or even very high [58] levels of antibiotic resistance in O157 *E. coli*, and so it is possible that antibiotic resistance in this group of *E. coli* is mediated by cryptic factors that are not represented in the determinants currently present in the CARD. Clearly, the parameters surrounding antibiotic resistance in this group of *E. coli* remain to be elucidated.

The factors in the accessory genome provide resistance against antibiotic classes that have activity against sensitive *E. coli*. The antimicrobials present in the CARD homologue model that we could not detect resistance factors for in *E. coli* are aminococumarins, triclosan, linezolids, elfamycins, fusidic acid, mupirocin and tunicamycin. Most of these antibiotics appear ineffective against *E. coli* owing to intrinsic insensitivity (aminocoumrin [46] and tunicamycin [59]), efficient efflux (linezolids [60] and fusidic acid [61]) or lack of penetration into the cell (elfamycins [62]). Furthermore, resistance towards mupirocin may be mediated through polymorphisms in the antibiotic target gene [63], which does not appear to be included in the CARD. It is only the antiseptic triclosan that these bacteria do not appear to have yet acquired defined resistance mechanisms, although wild strains of *E. coli* can vary in their sensitivity to this antimicrobial [64].

The genomes of *E. coli* that do encode resistance determinants are significantly more likely to contain multiple resistance than would be expected by chance. Indeed, one strain we identified as encoding resistance to 13 of the 18 classes of antibiotic that *E. coli* may resist. This strain was isolated from the rectum of a pig in China in 2012. Perhaps surprisingly, while it may be expected that this strain would originate within clades recognized for high levels of resistance, such as ST131, this strain belongs to phylogroup A. The massive compliment of AMRFs in this isolate include TEM-91, AAC-6′, ErmB, Oxa-31, CmlA4, FloR, Arr2, Sul2, RmtB, Aph-3′-Ib, MphA, Mrx, Aph-3’−1a, Aac-3-IV, Aac-6′-IB-cr, Sul3, Sul1, DfrA12, Mcr-1, OqxAB, CatB3 and Aph-4-Ia.

The trend for *E. coli* to accumulate AMR is preserved in the genomes for which the date of isolation has been recorded in GenBank. In many ways, our data showing that AMR has increased over time in *E. coli* may be unsurprising, as previous studies and meta-analyses of the published literature have shown that the frequency of specific antibiotic resistances and AMRs have been increasing over time in both *E. coli* and other Gram-negative bacilli [31, 65–71]. However, although it makes sense that a trend for increasing individual resistances would lead to increasing total numbers of resisted antibiotic classes, here we present genomic evidence that the historical increase in antibiotic resistance in *E. coli* is compounded to make contemporary strains more likely to be MDR strains than their ancestors. The implications of our analysis are dramatic – an average population level increase in resistance to an additional antibiotic every 20 years. *E. coli* isolated in 2016 are already, on average, resistant to almost three antibiotics, and our trendline predicts that the average *E. coli* isolated in 20 years are highly likely to be resistant to four agents. This observation supports the contention that high-level multidrug resistance will be an increasing challenge in healthcare over future decades.

Underlying this substantial increase in resistance may be the propensity for resistance determinants to co-associate within genomes, and our data indicates that AMR in *E. coli* are extensively interconnected. This is consistent with the co-carriage of resistances on plasmids and other mobile elements, although this facet was not a goal in our current investigation. This highly connected network of resistances in *E. coli* is extremely problematic, since it is indicative that treating a resistant infection with an antibiotic will not just result in the population level increase of resistance to the employed antibiotic, but will also result in the concomitant increase in the level of resistance to several other antibiotics. Furthermore, our visualization of how these relationships have developed over time show an explosion of co-resistances in the early years of the 21st century, and it may not be long before pan-resistance to antimicrobials becomes so frequent that no antibiotic will be useful against many infections. The recent report of a pan-resistant *Klebsiella* isolate [72] is a salient indication of the potential extent of risk. There is clearly an imperative to address this as indicated in numerous strategy documents from public health organizations and agencies.

Our findings suggest that incorporation of genomic epidemiology and modelling into selecting antibiotics for use in combination therapies presents opportunities to mitigate the spread of antibiotic resistance, as well as increase the probability for the infection to be cured. The use of combinatorial antibiotic therapy is controversial, and meta-analyses on the efficacy of these approaches often show no improvements in mortality rates compared with monotherapies [73–75], other than in high-risk patients [73] or where the infection was caused by *Pseudomonas aeruginosa* [74], which are characteristically multidrug resistant. Low-risk patients may even have an increased risk of death following combination therapy [73], perhaps due to the reported increase in the risk of complications such as nephrotoxicity [75]. Hence, careful consideration of clinical implications is a further significant issue.

Importantly, what may confound many meta-analyses is information on the appropriateness of the antibiotics used. Inappropriate antibiotic choices (which includes non-susceptibility and lack of timeliness) are associated with worse clinical outcomes, longer hospital stays and higher mortality than appropriate ones [76, 77]. Patients given inappropriate antibiotics can be three times less likely to survive hospitalization following Gram-negative sepsis than those treated appropriately [78]. Appropriate antibiotic choices require understanding of the AMR of individual case isolates, as well as the population genetics of AMR in the pathogen. Several of the meta-analyses showing poor results from combination therapy looked at combinations of β-lactams and aminoglycosides [74, 75], which we find are two of the most commonly resisted – and significantly likely to be resisted together – classes of antibiotic. Of the 2072 *E. coli* genomes that encode either β-lactam or aminoglycoside resistance over half of all strains – 80 % – encode both. It is possible that poor outcomes in these cases may have resulted from inappropriate antibiotic regimens rather than the failure of the combinatorial approach *per se*.

Indeed, our model reveals the potential futility of combining drugs such as β-lactams with aminoglycosides or fluoroquinolones, both for controlling infections and for reducing the spread of resistance genes – likely because of the high frequency that these antibiotics are currently co-resisted and in the presence of additional resistance genes – when resistant bacteria multiply following antibiotic challenge this causes the total number of resistance genes in the population to increase massively. Instead, we suggest that using information on the known diversity of AMR in *E. coli*, we should be choosing combinations of antimicrobials which are rarely, if ever, resisted together. We have identified 118 combinations of antibiotic classes where resistances to all three are not found in *E. coli –* many of these combinations have complementary targets and modes of action. By applying these combinations in our simulation, we can suggest that some of these combinations may both improve the number of successful treatments and slow the rate at which resistance genes spread in the *E. coli* population. However, we recognize that the combinations of antibiotics we have indicated may not be clinically practicable.

Combination therapies are not without risk. For example, it has been reported that combination antibiotics may select for broad-spectrum multi-drug resistance, at least in *P. aeruginosa* where dysregulated efflux pumps appear to drive high levels of resistance following ciprofloxacin (fluoroquinolone) and ceftazidime (β-lactam) treatment [79]. Hence, further evaluation of drug combinations will be necessary to inform any decision-making.

There is a wide diversity of *E. coli* in the GenBank database. However, and predictably considering the importance of AMR, several studies have sequenced genomes directly associated with resistance. Therefore, the sequenced *E. coli* population cannot be considered as representing a random sample of the wider population, and the risk of over-estimating AMR in *E. coli* should be interpreted considering this bias. However, our previous work has indicated that the representation of the diversity of *E. coli* in sequenced genomes is comprehensive [80], which may mitigate the effect of the bias caused by the selection of AMR strains for sequencing. Irrespective of how representative the 4021 *E. coli* subjected to this analysis are, the relentless emergence of multi-resistant strains will remain a major healthcare challenge for decades ahead.

## Data bibliography

1. Robert J Goldstone, Figshare https://dx.doi.org/10.6084/m9.figshare.4434776 (2016).
2. Robert J Goldstone, Figshare https://dx.doi.org/10.6084/m9.figshare.4434779 (2016).
3. Robert J Goldstone, Figshare https://dx.doi.org/10.6084/m9.figshare.4434782 (2017).
4. Robert J Goldstone, Figshare https://dx.doi.org/10.6084/m9.figshare.4434788 (2016).
5. Robert J Goldstone, Figshare https://dx.doi.org/10.6084/m9.figshare.4434794 (2016).
6. Robert J Goldstone, Figshare https://dx.doi.org/10.6084/m9.figshare.4434797 (2016).
7. Robert J Goldstone, Figshare https://dx.doi.org/10.6084/m9.figshare.4434800 (2016).
8. Robert J Goldstone, Figshare https://dx.doi.org/10.6084/m9.figshare.4595371 (2017).
9. Robert J Goldstone, Figshare https://dx.doi.org/10.6084/m9.figshare.4434803 (2017).
10. Robert J Goldstone, Figshare https://dx.doi.org/10.6084/m9.figshare.4434809 (2017).
11. Robert J Goldstone, Figshare https://dx.doi.org/10.6084/m9.figshare.4595389 (2017).
12. Robert J Goldstone, Figshare https://dx.doi.org/10.6084/m9.figshare.4595377 (2017).
13. Robert J Goldstone, Figshare https://dx.doi.org/10.6084/m9.figshare.4434815 (2017).
14. Robert J Goldstone, Figshare https://dx.doi.org/10.6084/m9.figshare.4434773 (2017).
15. Robert J Goldstone, Figshare https://dx.doi.org/10.6084/m9.figshare.4434818 (2017).
